# Regional and Vintage-Associated Differences in Commercial Chardonnay Wines from the Shandong Peninsula and the Chugoku Region: Physicochemical, Volatile, and Sensory Profiles with Exploratory Associations with Growing-Season Climate

**DOI:** 10.3390/foods15203627

**Published:** 2026-10-12

**Authors:** Dongyue Yang, Yang Yang, Ruirui Li, Xiaohong Tang, Ke Zhong, Yoshiyuki Murata, Fumio Fukuda, Zhiyu Li, Yuxia Sun

**Affiliations:** 1 Winegrape and Wine Technological Innovation Center of Shandong Province, Shandong Academy of Grape, Jinan 250100, China; yangdongyue1203@163.com (D.Y.); feixiang0507@126.com (Y.Y.); liruirui199103@163.com (R.L.); paper20190624@163.com (X.T.); zk17854117992@163.com (K.Z.); 2 Graduate School of Environmental and Life Science, Okayama University, Okayama 700-8530, Japan; muta@cc.okayama-u.ac.jp (Y.M.); ffukuda@okayama-u.ac.jp (F.F.)

**Keywords:** white wine, aroma, climate variability, comparative analysis, vintage variation

## Abstract

Integrated assessments of commercial Chardonnay wines from East Asia remain limited. This study compared wines from eight wineries—four in the Shandong Peninsula (China) and four in the Chugoku region (Japan)—across two vintages (2022–2023), with one bottle analyzed per winery–vintage combination, yielding 16 commercial wine observations. Physicochemical, HS-SPME-GC-MS volatile, sensory, and growing-season climate data were integrated. Among the sampled commercial wines, Chugoku wines generally showed higher acid retention and stronger floral–citrus characteristics, whereas Shandong wines were more fruit-forward, with higher fruity ester contributions in several 2023 samples. Exploratory climate–composition analysis identified a negative association between GDD and total terpenoids after FDR correction, whereas other associations did not remain significant after multiple-testing correction. These results indicate regional and vintage-associated differences among the sampled commercial wines. Because vinification practices and post-bottling factors were not fully controlled, climate–composition relationships should be interpreted as exploratory rather than causal.

## 1. Introduction

Global warming is widely regarded as a primary exogenous forcing on viticultural systems [1,2,3]. Higher ripening temperatures tend to bring harvest forward and shift wine composition in predictable ways—more alcohol, lower titratable acidity, higher pH, and changes in aroma profile and typicity [1,3,4]. Because interannual climatic variability within a region can exceed soil and other determinants for many quality attributes, cross-vintage comparisons are critical for robust inference [2,5]. Across climate gradients, warmer conditions generally favor sugar build-up and faster organic acid loss, whereas cooler ripening, especially under larger diurnal ranges, supports acid retention and may influence the accumulation of low-threshold volatiles and/or their precursors, thereby contributing to freshness and aromatic precision [2,3,4,6,7]. Water availability also affects berry growth, ripening, disease pressure, and the berry microenvironment, with subsequent effects on wine composition and aroma [2,3,8]. For this reason, climatic influences on wine quality are better considered together with compositional and sensory data rather than inferred from a single climatic variable.

Chardonnay is a globally emblematic white cultivar, widely grown and capable of marked stylistic plasticity across environments and vinification regimes [6,9,10]. It is generally regarded as a relatively neutral or weakly aromatic cultivar, and its final aroma profile is strongly shaped by both grape-derived compounds and fermentation metabolism [6,9]. Higher alcohols and their derived acetate esters, together with fatty acid ethyl esters, constitute much of the fruity–floral base; at moderate levels, higher alcohols can support ester formation and enhance aroma, but excessive levels may dampen aroma release and reduce complexity [6,11,12]. Varietal volatiles such as terpenes are often present at low concentrations, yet their low odor thresholds mean that small changes can still influence floral–citrus nuances and perceived elegance [7,13]. Regional and vintage-associated differences in Chardonnay therefore reflect the combined influence of environmental conditions, grape composition, fermentation, and other cellar practices rather than a single factor.

Within East Asia, the Shandong Peninsula in China and the Chugoku region in Japan provide an informative basis for comparison because they differ in geographical setting and growing-season conditions. Previous work has shown that Japanese Chardonnay wines can be differentiated according to geographical origin [14], while environmental conditions have also been linked to variation in Chardonnay grape and wine composition in other regions [7,10]. However, climatic conditions vary not only between regions but also among sites and vintages, and commercial wines may additionally differ in viticultural and winemaking practices. Thus, regional comparisons based on commercial wines should be interpreted as patterns among the sampled wines rather than as direct evidence of a climatic effect. In this context, growing-season climate indices can provide useful environmental information when interpreted together with physicochemical, volatile, and sensory data.

A number of studies have already combined chemical and sensory approaches to investigate the geographical differentiation of Chardonnay wines. Kustos et al. [15] characterized Australian Chardonnay wines of different provenance using sensory and chemical analyses, while Poggesi et al. [16] integrated multidimensional chemical profiling with sensory data to compare international commercial monovarietal Chardonnay wines. In China, Liu et al. [17] used a flavoromics approach to examine aroma characteristics of Chardonnay wines from the eastern foothills of the Ningxia Helan Mountain. More recent studies have extended this work by incorporating environmental information. Yang et al. [18] examined regional differences in wine aroma across several Chinese production regions, including Chardonnay, in relation to climatic factors. Yue et al. [19] investigated Chardonnay grapes and wines from different sub-regions and vintages in Ningxia using targeted metabolomics and environmental variables, whereas Hu et al. [20] combined volatile profiling, sensory evaluation, and terroir-related factors to assess sub-regional variation in Chardonnay. Together, these studies demonstrate the value of integrating chemical, sensory, and environmental information in Chardonnay research. Nevertheless, direct cross-country comparisons of commercial Chardonnay wines from major East Asian production areas across consecutive vintages remain limited.

Here, we analyzed commercial Chardonnay wines from two vintages (2022–2023), including four producers from the Shandong Peninsula and four from the Chugoku region. Physicochemical traits, volatile profiles, OAV-based aroma categories, and sensory attributes were examined to characterize regional and vintage-associated differences. Growing-season climatic indices were further compiled to explore their statistical associations with wine composition and sensory expression. Accordingly, this study aimed to describe regional and vintage-associated patterns within this set of commercial wines under a common analytical framework, rather than to make broader regional generalizations or infer direct climatic causality.

## 2. Materials and Methods

### 2.1. Wine Samples

Varietal Chardonnay wines were obtained from four wineries in the Shandong Peninsula (China) and four wineries in the Chugoku region (Japan) across two vintages (2022 and 2023), yielding 16 commercial wine observations. Commercial bottled wines were obtained through local retail channels or collaborating wineries. One bottle was analyzed for each winery–vintage combination; therefore, the 16 wines represent commercial wine observations rather than biological or production replicates. All analytical replicates were performed using aliquots from the same bottle and were used to assess analytical repeatability rather than bottle-to-bottle or lot-to-lot variation.

For each winery, the 2022 and 2023 samples corresponded to the same commercial label or wine style. Wine identity and grape origin were verified from retained bottle labels and available producer information. Lot numbers were available for the Hiroshima samples, while label production-date information was available for the Qingdao samples. Available information on fermentation, oak use, malolactic fermentation, and lees contact was collected from producer records and bottle information when available. Residual sugar was determined analytically for all wines. Because bottling dates were unavailable for most wines, the exact bottle age at the time of analysis could not be consistently determined. Therefore, differences between vintages were interpreted as vintage-associated differences rather than direct climatic vintage effects, because they may also reflect post-bottling evolution and winery-specific practices. Detailed wine-specific information, including commercial wine name, grape origin, lot/date information, and available vinification records, is provided in Table S1. Parameters that could not be verified were recorded as unavailable and were not interpreted as indicating the absence of a specific practice.

After acquisition, the bottles were stored at 4 °C in the dark until analysis.

### 2.2. Climate Data

Meteorological data for the Shandong Peninsula (China) and the Chugoku region (Japan) were collected for the 2022 and 2023 growing seasons from the Xihe-Energy database (http://xihe-energy.com), with ECMWF historical meteorological products selected as the data source. The dataset provided hourly records of air temperature and precipitation, which were aggregated into daily mean temperature, daily maximum and minimum temperatures, and daily precipitation before further calculation. The underlying ERA5 reanalysis product provides hourly meteorological data on a 0.25° × 0.25° regular latitude–longitude grid [21].

Climate data were extracted according to the documented grape-origin locations of the commercial wines rather than winery locations. The reference coordinates of the eight sampling sites (HRSM, OKYM, TOT, SHM, YTPL, YTLS, WHRS, and QDLX) are provided in Table S1. As exact vineyard coordinates were unavailable for some samples, the extracted data were considered representative of the corresponding grape-growing localities rather than vineyard-scale conditions. Therefore, climate data were interpreted as regional environmental information associated with the sampled wines.

Meteorological records were checked for completeness and abnormal values before calculation. Missing records, duplicated timestamps, and physically unrealistic values were examined during preprocessing, while meteorologically plausible extreme observations were retained.

Climate variables were calculated for annual, growing-season, and ripening periods. The growing season was defined as April–September, and August was selected as the ripening period for Chardonnay in the studied regions. Annual precipitation was calculated from January to December, whereas growing-season and ripening-period precipitation were calculated for April–September and August, respectively. Temperature-related variables, including mean temperature, maximum and minimum temperatures, and diurnal temperature range (DTR), were calculated for the corresponding periods.

Growing-season climatic variables and viticultural climate indices were calculated according to published definitions [22,23,24,25,26,27,28,29]. Growing degree days (GDD) were calculated using a base temperature of 10 °C [22,23]:

$$\mathrm{GDD}=\sum_{i=1}^{n}\max\left(T_{i}-10,0\right)$$

where *T_i_* represents the daily mean temperature during the growing season.

The Huglin Index (HI) was calculated according to Huglin [24]:

$$\mathrm{HI}=\sum \left[\frac{\left(T_{\mathrm{mean}}-10\right)+\left(T_{\max}-10\right)}{2}\right]\times k$$

where *T_mean_* and *T_max_* are the daily mean and maximum temperatures, respectively, and *k* is the latitude correction coefficient. Because all sampling sites were located below 40° N, *k* = 1.00 was applied.

The diurnal temperature range (DTR) was calculated as the difference between daily maximum and minimum temperatures. The cool night index was calculated as the mean minimum temperature during the ripening period following the viticultural climate classification proposed by Tonietto and Carbonneau [25]. The temperature range during ripening was calculated as the difference between the mean maximum temperature in September and the mean minimum temperature in August [26]. The continentality index, Branas hydrothermal index, and Selyaninov hydrothermal coefficient were calculated according to Rivas-Martínez et al. [27], Branas et al. [28], and Selyaninov [29], respectively.

All climatic variables and indices were calculated separately for each grape-origin locality and vintage. The resulting climate dataset was subsequently used to evaluate the associations between regional climatic conditions, wine physicochemical characteristics, volatile composition, and sensory attributes.

### 2.3. Basic Physicochemical Analysis

Ethanol, glucose, fructose, glycerol, and acetic acid concentrations were determined using an Agilent 1260 high-performance liquid chromatography system equipped with a refractive index detector (HPLC–RID; Agilent Technologies, Santa Clara, CA, USA), according to the method described by Yang et al. [30] with minor modifications. Wine samples were equilibrated to 20 ± 1 °C, thoroughly mixed, and filtered through 0.45 μm hydrophilic membranes before analysis. Chromatographic separation was performed on an Agilent Hi-Plex H column (300 × 7.7 mm, 8 μm) (Agilent Technologies, Santa Clara, CA, USA), maintained at 65 °C. Ultrapure water was used as the mobile phase at an isocratic flow rate of 0.6 mL min^−1^, with an injection volume of 20 μL. The compounds were quantified using external calibration curves constructed with the corresponding authentic standards under identical chromatographic conditions. Calibration curves were established for all quantified compounds, including ethanol, glucose, fructose, glycerol, and acetic acid, and showed good linearity. Detailed calibration information, including regression equations and coefficients of determination (R^2^), is provided in Table S2. Analytical precision was evaluated using repeated measurements of representative samples.

The pH values of wine samples were measured at 20 ± 1 °C using a pH meter (PHSJ-5, INESA Scientific Instrument Co., Ltd., Shanghai, China) after two-point calibration with standard buffer solutions (pH 4.00 and 7.00). Titratable acidity (TA) was determined by potentiometric titration with a standardized 0.06 M NaOH solution to an endpoint of pH 7.0 and expressed as g L^−1^ tartaric acid equivalents according to the OIV method [31].

Each wine was analyzed using technical replicates from the same bottle, and repeated measurements were used to assess analytical precision rather than treated as independent biological, production, or bottle replicates.

Ethanol content, pH, titratable acidity, and residual sugar are reported in Table 1, whereas glycerol and acetic acid concentrations, together with detailed commercial wine information, are provided in Table S1.

### 2.4. Volatile Analysis

Volatile aroma compounds in wines were analyzed by headspace solid-phase microextraction coupled with gas chromatography–mass spectrometry (HS-SPME–GC–MS; Agilent 7890 GC–5975 MSD, Agilent Technologies, Santa Clara, CA, USA) following Li et al. [32] with minor modifications. Briefly, 8 mL of wine, 2 g of NaCl, and 20 μL of 4-methyl-2-pentanol (2.00 g L^−1^) as the internal standard were placed in a 20 mL vial. After equilibration at 45 °C for 10 min, volatile compounds were extracted using a 50/30 μm DVB/CAR/PDMS fiber (Agilent Technologies, Santa Clara, CA, USA) at 45 °C for 50 min and desorbed at 250 °C for 10 min. Separation was performed on a DB-WAX column (Agilent Technologies, Santa Clara, CA, USA), 30 m × 0.32 mm × 0.25 μm, using helium at 0.8 mL min^−1^. The oven temperature was programmed from 40 °C to 45 °C at 1 °C min^−1^, then to 180 °C at 3 °C min^−1^, and finally to 230 °C at 5 °C min^−1^. The MS was operated in EI mode at 70 eV with a scan range of m/z 35–450.

Volatile compounds were identified by comparison with the NIST20.L spectral library, calculated retention indices (RIs), and corresponding authentic standards analyzed under the same chromatographic conditions. Spectral matching with NIST20.L was used as an additional confirmation criterion. Quantification was performed using external calibration curves prepared with the corresponding authentic standards in a 12% (*v*/*v*) ethanol matrix. All calibration curves showed good linearity (R^2^ > 0.98). Regression equations, linear ranges, R^2^ values, RI data, and identification information are provided in Table S3. Because authentic standards were available for all compounds, no semi-quantification using structural analogues was applied.

Limits of detection (LOD) and quantification (LOQ) were determined at signal-to-noise ratios of 3 and 10, respectively. Intra-day and inter-day precision were expressed as relative standard deviations (RSDs), and recovery was evaluated by standard addition to representative wine samples. Detailed validation results are provided in Table S4.

### 2.5. Calculation of Odor Activity Values

The contribution of specific volatiles to overall wine aroma was assessed through the calculation of Odor Activity Values (OAVs). Specifically, each OAV was computed by dividing the quantified concentration of an aroma compound by its olfactory threshold value in the same concentration unit. Odor thresholds and their literature sources are provided in Table S3. Compounds with OAV ≥ 0.1 were considered potentially odor-active.

For the aroma-category analysis, volatile compounds were assigned to six sensory categories—fruity, floral, caramel, fatty, herbaceous, and chemical—according to their reported odor descriptors. When a compound was associated with more than one descriptor, its predominant descriptor was used for category assignment to avoid double counting. The same category assignment was applied consistently to all wine samples. The OAVs of all compounds assigned to the same sensory category were summed to obtain the corresponding category OAV for each wine. No scaling or normalization was applied after summation; therefore, the values displayed in the radar plots represent the original summed OAVs for each sensory category.

### 2.6. Sensory Evaluation

Quantitative descriptive analysis (QDA) was performed by a trained panel of 16 experienced tasters (4 males and 12 females, aged 27–49 years) under controlled conditions (20 ± 2 °C and 60–70% relative humidity) following established sensory evaluation principles [33]. Panel preparation consisted of one descriptor-selection session followed by two 1 h training sessions. During training, panelists discussed descriptor definitions and reference materials, including rose water/green tea (flowery), lemon/grapefruit (citric), mango/pineapple (tropical fruit), peach/pear (sweet fruit), almond (nutty), sucrose solution (sweetness), lactic acid solution (lactic), toasted bread/oak (toasted), wet stone/flint (mineral), and green bell pepper/grass (herbaceous). Panel consistency was evaluated based on descriptor recognition and rating repeatability before formal evaluation. All participants provided informed consent before the sensory evaluation.

Wine samples (~30 mL) were served at 10–12 °C in coded ISO tasting glasses. Samples were presented blindly using randomized three-digit codes, and the evaluation order was balanced among panelists. Each wine was evaluated in triplicate, with water and unsalted crackers provided for palate cleansing between samples. Each attribute was rated on a nine-point scale ranging from 1 (extremely low intensity) to 9 (extremely high intensity), with 5 representing moderate intensity.

Sensory data were analyzed using a linear mixed-effects model. Wine sample and evaluation session (replicate) were included as fixed effects, panelist was included as a random effect, and repeated evaluations of wines were modeled within panelist. This structure accounted for the non-independence of repeated ratings provided by the same assessor across replicate sessions. Tukey-adjusted estimated marginal means were used for post hoc pairwise comparisons when significant effects were detected.

### 2.7. Statistical Analysis

Statistical analyses were conducted using Microsoft Excel 2019, SPSS 22.0, GraphPad Prism 14.0 software (GraphPad Software, San Diego, CA, USA), and Metware Cloud (https://cloud.metware.cn). Data are presented as mean ± standard deviation (SD), and differences were considered significant at *p* < 0.05.

Because the same wineries were sampled across two vintages, linear mixed-effects models were applied for region and vintage comparisons, with region, vintage, and their interaction as fixed effects, and winery nested within region as a random effect. Vintage was treated as a repeated factor within winery. Tukey-adjusted estimated marginal means were used for post hoc comparisons, and effect sizes with 95% confidence intervals were calculated where applicable.

For sensory data, the linear mixed-effects model described in Section 2.6 was applied, with wine sample and evaluation session (replicate) as fixed effects, panelist as a random effect, and repeated evaluations modeled within panelist. Tukey-adjusted estimated marginal means were used for post hoc comparisons where appropriate.

PCA, heatmap visualization, and Pearson correlation analysis were performed to explore relationships among wine composition, sensory attributes, and climatic indices. For the climate–composition analysis, Pearson correlations were restricted a priori to four representative climatic variables and six wine-composition variables. *p*-values from the 24 correlations were adjusted using the Benjamini–Hochberg false discovery rate (FDR) procedure, with q < 0.05 considered significant. Potentially influential observations in the climate–composition correlations were screened using leverage and Cook’s distance from the corresponding simple linear models. Observations with leverage > 2p/n or Cook’s distance > 4/n were flagged for further inspection. Robustness of the principal associations was further assessed by leave-one-out sensitivity analysis, in which each of the 16 winery–vintage observations was sequentially omitted, and Pearson’s r was recalculated.

## 3. Results and Discussion

### 3.1. Climate Variability Between Regions and Vintages

**Figure 1:**
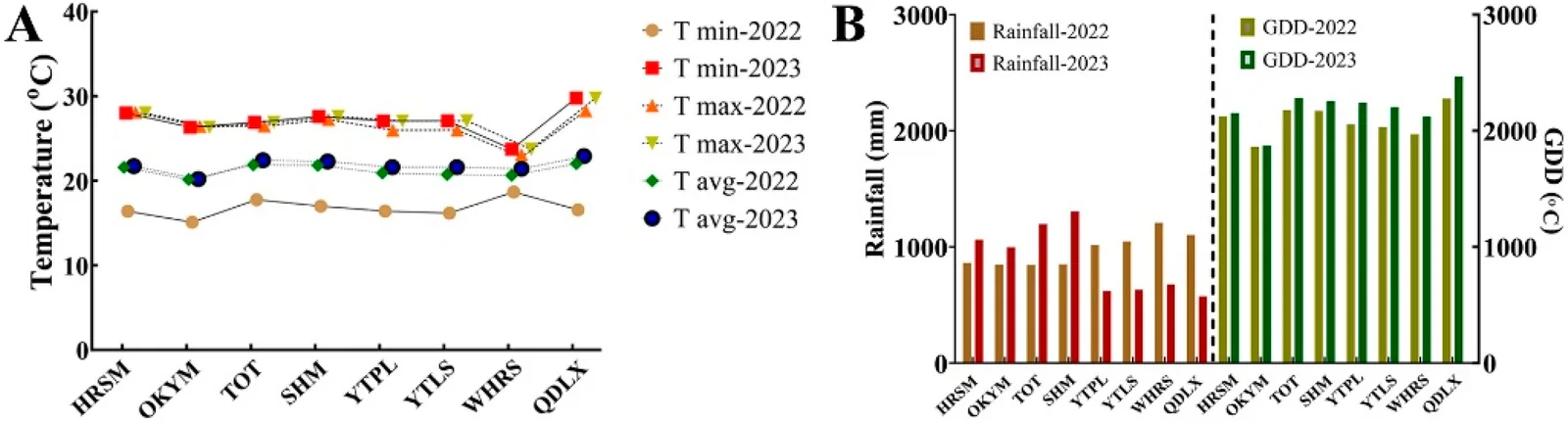
Comparison of growing-season climatic indices at different sampling sites in the Shandong Peninsula and the Chugoku region in Japan during 2022 and 2023: (**A**) Comparison of growing-season minimum temperature (T min), maximum temperature (T max), and average temperature (T avg) across sampling sites. (**B**) Comparison of growing-season rainfall and growing degree days (GDD) across sampling sites; the left y-axis indicates rainfall (mm), and the right y-axis indicates GDD (°C).

April–September climate showed contrasting regional and vintage-associated patterns. Shandong Peninsula sites were systematically warmer than those in the Chugoku region (higher T avg and GDD), and 2023 was warmer than 2022 in both regions (T avg/GDD increased) (Figure 1A). In contrast, moisture trends diverged: 2023 precipitation increased in the Chugoku region but declined sharply in the Shandong Peninsula; specifically, SHM recorded higher rainfall in 2023, whereas YTPL and QDLX exhibited pronounced decreases (Figure 1B). These differences are consistent with previous evidence that temperature and water availability can influence grape development, metabolite accumulation, and wine composition [2,3,6,8]. From an interpretive perspective, warmer ripening conditions are generally associated with earlier harvest, higher alcohol, lower acidity, and shifts in wine aroma profiles, whereas cooler conditions are often related to stronger acid retention and differences in aroma development [1,3,4,6]. Taken together, the contrasting warm–dry and warm–wet patterns observed in 2023 provide climatic context for interpreting the regional and vintage-associated differences in wine composition. These interpretations should be considered as climate-associated tendencies rather than direct causal effects [2,3,4,6].

### 3.2. Physicochemical Characteristics

Across the key oenological indices, ethanol, titratable acidity (TA), and pH showed regional and vintage-associated variation (Table 1). Overall, Chugoku wines were characterized by lower pH and higher TA, whereas wines from the Shandong Peninsula showed higher pH and lower TA, indicating differences in acid-related characteristics among the sampled commercial wines. Across vintages, most 2023 samples displayed higher pH and lower TA, together with increased ethanol in several Shandong Peninsula wines. These vintage-associated differences may reflect the combined influence of growing-season conditions, post-bottling evolution, and winery-specific production decisions. Previous work has shown that viticultural and fermentation strategies can alter wine ethanol and acidity, highlighting the contribution of management practices to these compositional traits [34]. Additional physicochemical parameters, including residual sugar, glycerol, and acetic acid, are summarized in Table S1. Together, these physicochemical contrasts provide a useful compositional basis for interpreting volatile outcomes, because ethanol and acidity/pH can influence fermentation kinetics, metabolic flux allocation related to higher alcohol and ester biosynthesis, and aroma expression through changes in volatile solubility, partitioning, and headspace release dynamics [3,6,11].

### 3.3. Volatile Aroma Composition

**Figure 2:**
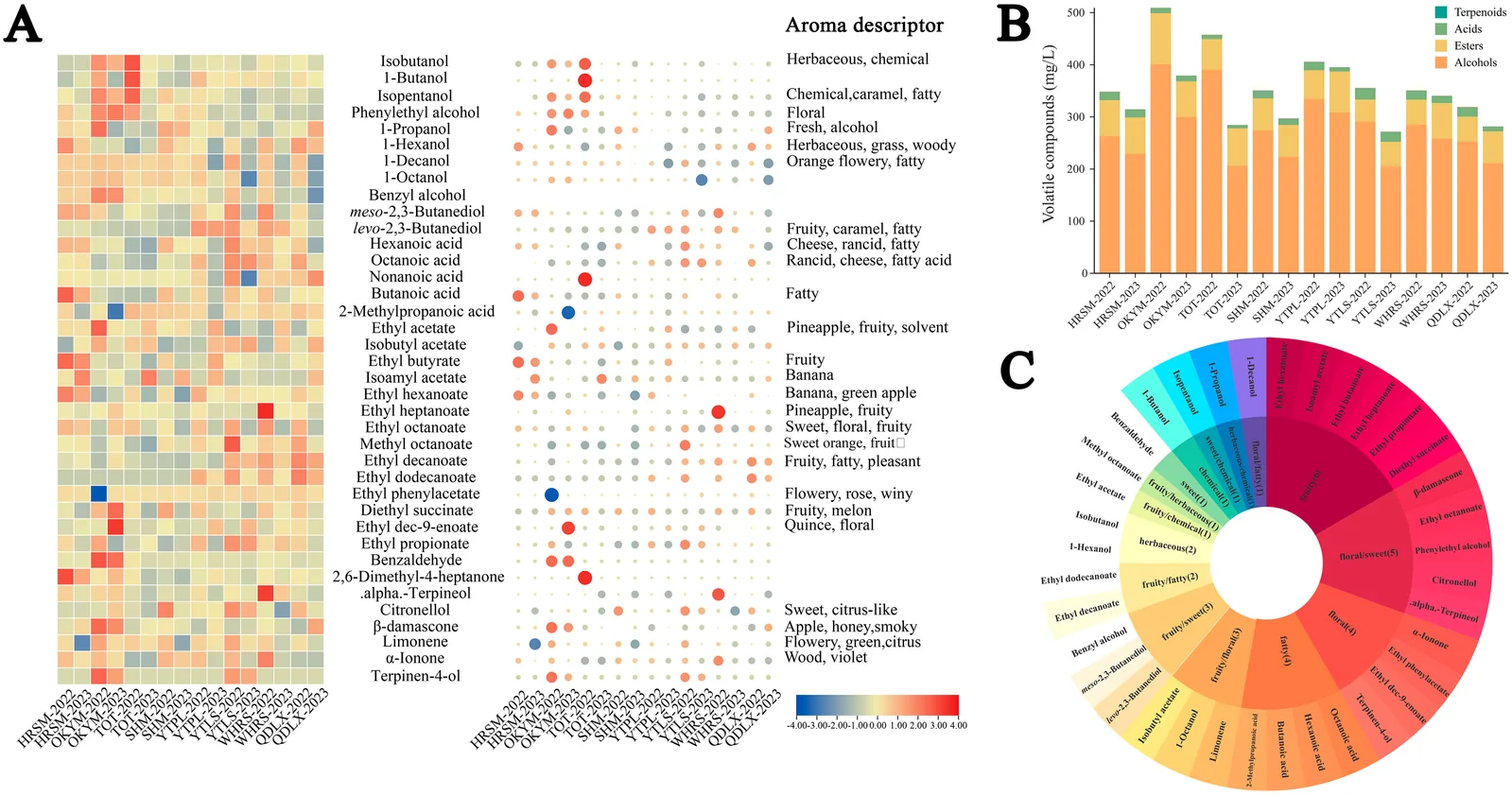
Volatile composition and sensory-related characteristics of Chardonnay wines from the Shandong Peninsula and the Chugoku region of Japan: (**A**) Standardized heatmap of 38 volatile compounds across samples and a bubble plot based on compounds with *OAV* ≥ 0.1. (**B**) Stacked bar chart of the total concentrations of volatile compounds (mg/L), grouped into alcohols, esters, volatile fatty acids, and terpenoids. (**C**) Sunburst plot summarizing the sensory attributes of key volatile compounds.

A total of 38 volatile compounds were quantified in Chardonnay wines from the Shandong Peninsula (China) and the Chugoku region (Japan). Overall, the volatile profiles were dominated by alcohols, esters, and volatile fatty acids. In contrast, varietal aroma compounds, particularly terpenes, accounted for only a minor fraction, with concentrations consistently remaining well below 1 mg/L (Figure 2B). This compositional pattern aligns with the general aromatic characteristics of white wines, in which the aroma backbone is largely formed by yeast-derived metabolites generated during alcoholic fermentation, while varietal character compounds are typically present only at low concentrations [6,11,12,13,35,36].

Total volatile concentrations ranged from 275.4 to 367.9 mg/L in the Shandong Peninsula wines and from 274.3 to 489.3 mg/L in the Chugoku wines. Notably, OKYM-2022 (489.3 mg/L) and TOT-2022 (456.7 mg/L) showed the highest total volatile concentrations, whereas TOT-2023 (274.3 mg/L) represented the lowest (Figure 2B). Both regions displayed a typical fermentation-driven aroma profile, characterized by alcohols (71.3–85.8%), esters (12.8–26.2%), and volatile fatty acids (1.5–7.0%). While wine aroma is a complex ensemble of grape-derived precursors and fermentation- or aging-induced transformations, only a subset of these volatiles reaches concentrations of direct sensory relevance [6,9,11,13,37]. The fruity character, for instance, is shaped by the synergistic effects of acetate esters, ethyl esters, and higher alcohols, compounds whose accumulation is highly sensitive to yeast strain selection, nitrogen availability, and thermal conditions during fermentation [6,11,12,35,36]. More broadly, our data suggest that while fermentation dictates the quantitative framework, the final aroma expression is fine-tuned by the interplay of varietal background, environmental factors, and viticultural management [2,3,6,13,38,39].

To further characterize inter-sample variation in individual compounds and their potential sensory relevance, a normalized heatmap together with a bubble plot based on compounds with OAV ≥ 0.1 was used for visualization (Figure 2A). The heatmap showed marked inter-sample variation in volatile composition. The sunburst plot further indicated that selected volatiles were associated with chemical, floral–sweet, fatty, and fruity–green sensory attributes, together with herbaceous, citrus-like, and violet/floral nuances (Figure 2C). The differences between wines from the two regions were reflected not only in the overall abundance of volatile compounds but also in the composition and relative weighting of key odor-active constituents.

#### 3.3.1. Alcohols

Alcohols were the dominant volatile class in all wines, with total concentrations ranging from 208.1 to 296.5 mg/L in the Shandong Peninsula samples and from 195.6 to 391.7 mg/L in those from the Chugoku region (Figure 2B). Isoamyl alcohol was the predominant compound in both regions. However, the relative abundance patterns of secondary alcohols differed among the sampled wines from the two regions: Chugoku wines were characterized mainly by higher 1-propanol, isobutanol, and 2-phenylethanol levels, whereas levo-2,3-butanediol was more prominent in the Shandong Peninsula wines (Figure 2A). These higher alcohols are mainly produced by yeast through central carbon metabolism and the Ehrlich pathway via the catabolism of branched-chain and aromatic amino acids, including valine, leucine, isoleucine, and phenylalanine [6,11,36].

In white wines, higher alcohols form an important part of the fermentative aroma background and also act as precursors for acetate ester formation [6,11,37]. Their sensory effects depend largely on concentration: moderate levels may enhance aromatic intensity and contribute to fruity or floral expression, whereas excessive accumulation may suppress aroma release and reduce aromatic finesse and complexity [6,9,11,35].

At the regional level, the Chugoku wines were characterized not only by higher isoamyl alcohol levels in several samples but also by generally higher concentrations of 2-phenylethanol. Given the known floral character of 2-phenylethanol, this pattern was consistent with a relatively more pronounced floral background in the Chugoku wines [6,9,11]. By contrast, several Shandong wines, particularly YTPL-2022/2023, YTLS-2022/2023, and WHRS-2022/2023, showed markedly higher levo-2,3-butanediol concentrations (21.8–76.6 mg/L), suggesting a different compositional allocation within the alcohol fraction (Figure 2B). Overall, the alcohol fraction showed substantial inter-sample variation, with different relative contributions of higher alcohols among the sampled wines from the two regions.

#### 3.3.2. Esters

Esters were the second most abundant volatile class in all wines and represented a major group contributing to sensory differentiation among samples (Figure 2A–C). Quantitatively, total ester concentrations were generally higher in the Chugoku wines (58.3–97.6 mg/L) than in the Shandong Peninsula wines (42.7–78.6 mg/L), and the Chugoku wines also showed greater inter-sample variation.

At the compound level, acetate esters were the dominant ester subclass. Ethyl acetate was the most abundant individual ester, ranging from 30.2 to 69.2 mg/L in the Shandong wines and from 47.7 to 89.7 mg/L in the Chugoku wines. As an important contributor to the youthful fruity and floral character of white wines, its sensory role is concentration-dependent [6,11,35,36]. OKYM-2022 showed the highest ethyl acetate concentration, contributing substantially to its higher total ester abundance.

Isoamyl acetate varied markedly among wines (0.1–5.0 mg/L), with higher concentrations observed in several 2023 samples, particularly TOT-2023, HRSM-2023, SHM-2023, and YTPL-2023. Given its characteristic banana- and sweet-fruit-like aroma, this compound may have contributed to the fruity sensory expression of these samples [6,11,13,35,37]. Fatty acid ethyl esters provided an important chemical basis for fruity expression in Chardonnay wines [6,11,13,17,40]. Ethyl hexanoate and ethyl octanoate were consistently detected at relatively high levels and are associated with apple-, pear-, and tropical-fruit-like aroma notes [6,13,37].

Regional and vintage comparisons further indicated contrasting patterns in ester expression. In the Shandong wines, total ester concentrations were higher in 2023 (mean: 63.3 mg/L) than in 2022 (mean: 48.4 mg/L), indicating a stronger ester contribution in the later vintage. By contrast, the Chugoku wines showed a slight decline in total esters in 2023 relative to 2022, which may reflect differences in wine composition, fermentation-related factors, and post-bottling evolution among the sampled commercial wines [6,9,11]. Taken together, the observed differences between the two groups of commercial wines were reflected not only in total ester abundance but also in the relative contributions of individual odor-active esters.

#### 3.3.3. Acids

Acids were the third most abundant class of volatile compounds, with total concentrations ranging from 7.9 to 22.1 mg/L in the Shandong wines and from 6.6 to 16.0 mg/L in the Chugoku wines (Figure 2B). This fraction was dominated by octanoic and hexanoic acids, with lower levels of butanoic, decanoic, and nonanoic acids also detected (Figure 2A). The formation of these compounds is closely associated with must composition, yeast lipid metabolism, and fermentation conditions [6,11,40].

From a sensory perspective, medium-chain fatty acids (C_6_–C_10_) may contribute positively to aroma complexity at moderate concentrations, whereas elevated levels are more often associated with fatty, rancid, or cheesy notes [6,11,40]. In the present study, several samples, including YTLS-2022 from the Shandong Peninsula, showed total volatile fatty acid concentrations above 20 mg/L. However, because sensory impact depends on the concentrations and odor thresholds of individual compounds rather than on the summed fatty acid concentration alone, this result was interpreted primarily as a compositional difference rather than direct evidence of stronger fatty sensory perception.

In addition to their direct sensory relevance, volatile fatty acids are also closely related to the formation of fatty acid ethyl esters, and variation in their abundance may therefore affect fruity ester expression [6,11,40]. At the regional level, the Shandong wines generally showed a slightly higher contribution of volatile fatty acids than the Chugoku wines, although this pattern varied across sites and vintages. For example, the TOT samples consistently exhibited relatively low fatty acid levels in both vintages, suggesting a comparatively weaker fatty acid contribution at this site. Overall, although volatile fatty acids were not the dominant contributors to the aroma profile, they may have influenced aroma composition both directly and through their role as precursors of fatty acid ethyl esters [6,11].

#### 3.3.4. Terpenoids

Terpenes and norisoprenoids were the least abundant volatile group in all wines, with total concentrations of approximately 0.056–0.097 mg/L (Figure 2B). Although their absolute concentrations were much lower than those of fermentation-derived volatiles, their sensory relevance cannot be evaluated on the basis of concentration alone, because compounds such as α-terpineol, citronellol, β-damascenone, and α-ionone are characterized by very low odor thresholds and may therefore contribute to aroma even at relatively low concentrations [6,9,13,37,41]. In general, these grape-derived or varietal-associated compounds contribute floral, citrus, honey-like, and violet-like nuances and may influence the aromatic complexity of Chardonnay wines [6,13,37,41].

Compared with the Shandong Peninsula wines, the Chugoku wines tended to show a relatively stronger floral pattern among the samples analyzed. This pattern was consistent, on the one hand, with higher levels of 2-phenylethanol, a compound associated with floral aroma [6,9,11,35], and, on the other hand, with sample-specific differences in low-threshold terpenes and norisoprenoids (Figure 2A).

Overall, the volatile profiles of all wines were characterized by a typical fermentation-driven aromatic framework, in which alcohols and esters formed the main quantitative basis of the volatile profile, while volatile fatty acids, terpenes, and norisoprenoids made smaller but potentially sensory-relevant contributions [6,11,13,37,41]. Among the sampled commercial wines, the Chugoku wines tended to show relatively stronger floral/citrus nuances, whereas the Shandong wines were more fruit-forward, particularly in the 2023 vintage. Vintage-associated differences were mainly reflected in changes in the relative distribution of fermentation-derived compounds, especially esters and fatty acids, rather than in any fundamental shift in the overall aromatic framework [6,11,13].

### 3.4. Sensory Profile Differences

**Figure 3:**
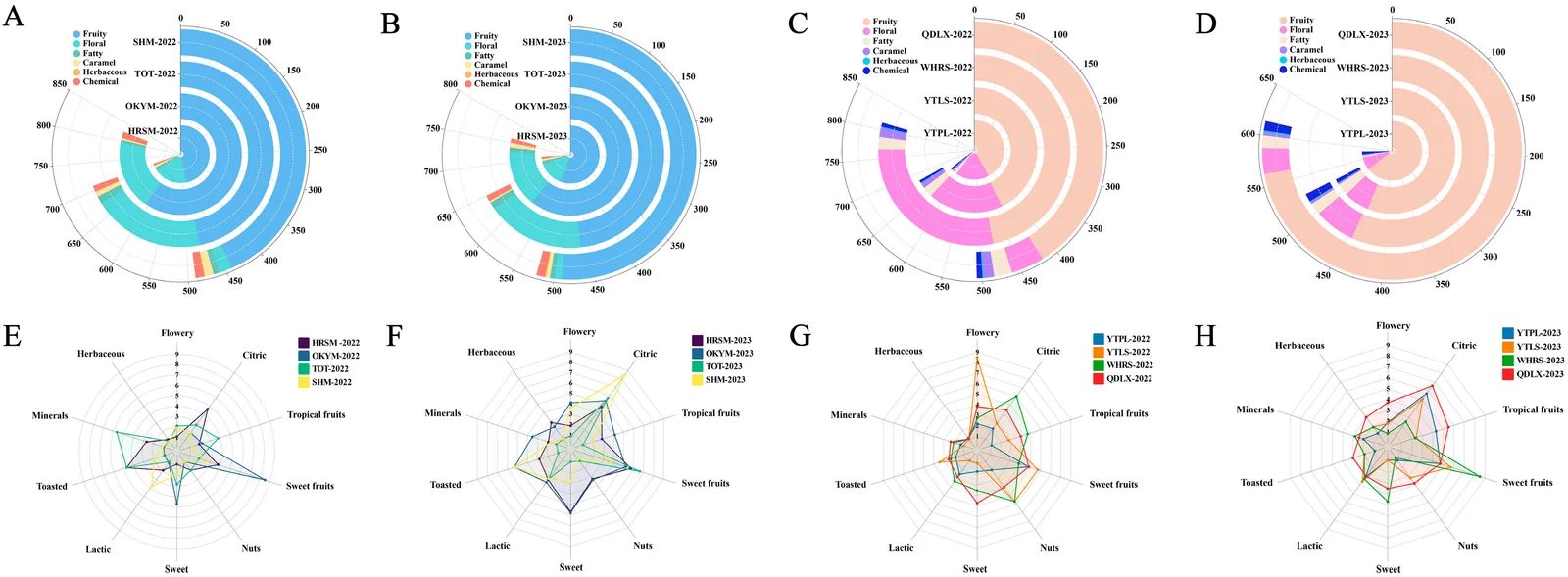
Aroma-category distribution and sensory radar analysis of Chardonnay wines from the Shandong Peninsula and the Chugoku region of Japan: (**A**–**D**) Circular plots showing the summed OAVs of the six aroma categories: (**A**) Chugoku wines from the 2022 vintage; (**B**) Chugoku wines from the 2023 vintage; (**C**) Shandong Peninsula wines from the 2022 vintage; (**D**) Shandong Peninsula wines from the 2023 vintage. (**E**–**H**) Radar plots of sensory descriptor intensities, including flowery, citric, tropical fruits, sweet fruits, nuts, sweet, lactic, toasted, mineral, and herbaceous notes: (**E**) Chugoku wines from the 2022 vintage; (**F**) Chugoku wines from the 2023 vintage; (**G**) Shandong Peninsula wines from the 2022 vintage; (**H**) Shandong Peninsula wines from the 2023 vintage.

Radar plots and intensity ratings for the ten sensory attributes revealed regional and vintage-associated variation in Shandong and Chugoku Chardonnay wines (Figure 3E–H). Chugoku wines generally showed higher citric intensities and, in some cases, stronger toasted and sweet impressions, whereas Shandong Peninsula wines were more fruit-forward, with higher sweet-fruit and tropical-fruit intensities. These differences may also reflect cellar practices: oak contact can enhance toasted notes, malolactic fermentation and lees/bâtonnage can influence lactic or creamy characteristics, yeast strain can affect fermentation-derived aroma, and residual sugar can contribute to perceived sweetness [6,11,42]. Such technological factors may be more proximal drivers of sensory expression than climate indices.

These sensory differences were broadly consistent with the patterns observed in the OAV-based plots (Figure 3A–D), which summarized the OAVs of compounds assigned to six aroma categories (fruity, floral, caramel, fatty, herbaceous, and chemical). The OAV plots indicated that fruity OAV was the dominant contributor across all samples, with Shandong wines generally showing higher fruity and fatty category OAVs, whereas several Chugoku wines showed relatively stronger floral contributions.

Across vintages, 2023 samples tended to show higher sweetness and toastiness scores, although these differences may reflect a combination of vintage conditions, wine composition, and post-bottling evolution. Overall, the sensory patterns were broadly consistent with the volatile-composition and OAV results, suggesting that fruit and floral attributes may reflect the combined contribution of multiple odor-active compounds [6,9,11,13,37,41].

### 3.5. Regional and Vintage-Associated Differentiation

**Figure 4:**
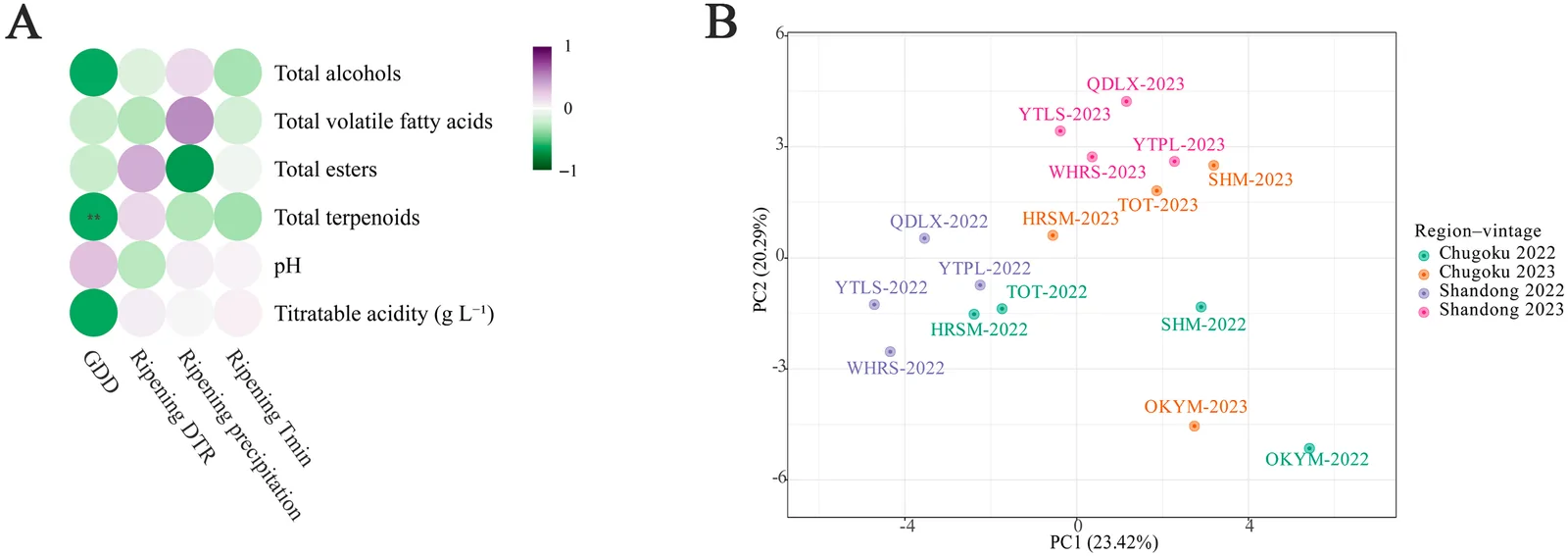
Exploratory climate–composition associations and multivariate variation among commercial Chardonnay wines: (**A**) Pearson correlation analysis between four a priori selected climatic variables and six major wine-composition variables based on 16 winery–vintage observations. Color indicates the direction and magnitude of Pearson’s correlation coefficient (r). *p*-values were adjusted using the Benjamini–Hochberg false discovery rate procedure. ** *q* < 0.01. Corresponding correlation coefficients, 95% confidence intervals, raw *p*-values, and FDR-adjusted q-values are provided in Table S5. (**B**) PCA score plot based on the 38 quantified volatile compounds in the 16 commercial Chardonnay wine observations. PC1 and PC2 explained 23.42% and 20.29% of the total variance, respectively. Colors indicate region–vintage groups.

Pearson correlation analysis based on 16 winery–vintage observations was used to explore associations between four a priori selected climatic variables and six major wine-composition variables (Figure 4A). To reduce redundancy among collinear climatic indices, the analysis focused on GDD, ripening-period mean DTR, ripening-period precipitation, and ripening-period mean minimum temperature. After Benjamini–Hochberg FDR correction, only the negative association between GDD and total terpenoids remained statistically significant (r = −0.781, q = 0.0086). Negative correlations of GDD with total alcohols and titratable acidity, and of ripening-period precipitation with total esters, were observed at the unadjusted level but did not remain significant after FDR correction. Influence diagnostics identified several observations with elevated leverage; however, no single observation accounted for the FDR-supported GDD–total terpenoids association. In leave-one-out analyses, this correlation remained negative in all 16 iterations (r = −0.83 to −0.72; all raw *p* < 0.003). Other unadjusted associations showed greater sensitivity to individual observations and were therefore interpreted as exploratory. These patterns may also reflect differences in bottle age, post-bottling evolution, and winery-specific practices that could not be fully controlled [2,3,6,13].

PCA based on the 38 quantified volatile compounds was used as an unsupervised approach to visualize overall variation among the 16 commercial wine observations (Figure 4B). PC1 and PC2 explained 23.42% and 20.29% of the total variance, respectively. The score plot showed substantial overlap between the two regional groups, while several wineries exhibited noticeable between-vintage displacement. The magnitude and direction of these shifts varied among wineries, indicating that the observed vintage-associated variation was not uniform across producers. Given the limited sample size, the PCA patterns were interpreted descriptively and were not considered evidence of predictive regional classification.

Overall, the correlation and PCA results illustrate regional and vintage-associated variation within the sampled commercial wines, but they should not be interpreted as independent evidence of climatic causality or broad regional differentiation [14,15,16,17,18,19,20,38,39,43].

## 4. Conclusions

Using physicochemical, volatile, and sensory data from 16 commercial Chardonnay wines across two vintages and regions, this study identified regional and vintage-associated differences among the sampled wines. Chugoku wines generally showed higher TA, lower pH, and stronger floral–citrus characteristics, whereas Shandong Peninsula wines were more fruit-forward with greater contributions from selected fruity esters. These findings describe patterns within the sampled commercial wines rather than broader regional characteristics. Because vinification practices, bottle age, and post-bottling evolution were not fully controlled, associations between climate indices and wine composition should be interpreted as exploratory rather than causal. Further studies using standardized vinification and vineyard-level meteorological data are needed to clarify the relative contributions of climate and winemaking practices.

## Figures and Tables

**Table 1:** Commercial wine information, grape origins, and physicochemical characteristics of Chardonnay wines analyzed in this study.

Site (Code)	Wine Producer	Commercial Wine	Grape Origin	Vintage	pH	TA (g L^−1^)	Ethanol (% *v*/*v*)	Residual Sugar (g L^−1^)
Hiroshima (HRSM)	Hiroshima Miyoshi Winery	TOMOÉ Chardonnay Crisp	Miyoshi, Hiroshima, Japan	2022	3.36 ± 0.03	6.60 ± 0.35	11.88 ± 0.22	0.98 ± 0.06
		TOMOÉ Chardonnay Crisp		2023	3.41 ± 0.03	6.55 ± 0.22	12.34 ± 0.02	1.41 ± 0.05
Okayama (OKYM)	Domaine Tetta	Domaine Tetta Chardonnay d’Or	Niimi, Okayama, Japan	2022	3.38 ± 0.04	6.81 ± 0.14	11.49 ± 0.04	1.14 ± 0.08
		Domaine Tetta Chardonnay d’Or		2023	3.40 ± 0.05	6.40 ± 0.32	12.90 ± 0.09	0.99 ± 0.04
Tottori (TOT)	Kurayoshi Winery	Hakushu Chardonnay	Tottori, Japan	2022	3.41 ± 0.03	6.67 ± 0.23	13.72 ± 0.03	0.89 ± 0.02
		Hakushu Chardonnay		2023	3.42 ± 0.15	6.53 ± 0.19	12.50 ± 0.21	1.19 ± 0.09
Shimane (SHM)	Oku-Izumo Vineyard	Oku-Izumo Wine Chardonnay Unwooded	Shimane, Japan	2022	3.45 ± 0.11	6.40 ± 0.35	12.31 ± 0.02	0.90 ± 0.03
		Oku-Izumo Wine Chardonnay Unwooded		2023	3.37 ± 0.05	6.27 ± 0.54	12.23 ± 0.16	1.52 ± 0.01
Penglai, Yantai (YTPL)	COFCO GreatWall Wine (Penglai) Co., Ltd.	GreatWall Chardonnay Dry White Wine	Penglai, Yantai, Shandong, China	2022	3.44 ± 0.12	6.50 ± 0.55	11.82 ± 0.28	0.98 ± 0.07
		GreatWall Chardonnay Dry White Wine		2023	3.53 ± 0.03	6.02 ± 0.25	12.12 ± 0.12	0.79 ± 0.11
Laishan, Yantai (YTLS)	Pula Valley	Pula Valley Chardonnay Dry White Wine	Yantai, Shandong, China	2022	3.49 ± 0.04	6.52 ± 0.28	10.80 ± 0.11	1.12 ± 0.06
		Pula Valley Chardonnay Dry White Wine		2023	3.65 ± 0.14	5.88 ± 0.24	12.39 ± 0.24	0.75 ± 0.07
Rushan, Weihai (WHRS)	Taiyihu Winery	Taiyihu Chardonnay Dry White Estate Wine	Weihai, Shandong, China	2022	3.40 ± 0.06	6.40 ± 0.47	10.75 ± 0.26	1.06 ± 0.01
		Taiyihu Chardonnay Dry White Estate Wine		2023	3.67 ± 0.03	6.07 ± 0.31	12.04 ± 0.61	0.84 ± 0.04
Laixi, Qingdao (QDLX)	Chateau Nine Peaks	Chateau Nine Peaks Chardonnay Classic	Qingdao, Shandong, China	2022	3.39 ± 0.14	6.27 ± 0.42	11.15 ± 0.33	0.94 ± 0.06
		Chateau Nine Peaks Chardonnay Classic		2023	3.55 ± 0.06	5.73 ± 0.12	12.69 ± 0.11	0.87 ± 0.02

**Note:** Values are expressed as mean ± SD of analytical replicates from the same bottle. One bottle was analyzed for each winery–vintage combination; therefore, analytical replicates were not considered independent biological, production, or bottle replicates. Statistical comparisons were performed using linear mixed-effects models with winery as a random effect. Detailed information on lot/date information and available vinification practices is provided in Table S1. Bottle age was not calculated consistently because bottling dates were unavailable for most wines.

## Data Availability

The original contributions presented in this study are included in the article/Supplementary Materials. Further inquiries can be directed to the corresponding authors.

## Supplementary Materials

The following supporting information can be downloaded at: https://www.mdpi.com/article/10.3390/foods15203627/s1, Table S1: Commercial wine information and additional physicochemical characteristics of Chardonnay wines from the Shandong Peninsula and the Chugoku region; Table S2: Calibration parameters of compounds determined by HPLC-RID in Chardonnay wines; Table S3: Calibration parameters, identification information, and odor thresholds of volatile compounds in Chardonnay wines; Table S4: Analytical validation parameters of volatile compounds determined by HS-SPME-GC-MS; Table S5: Pearson correlation analysis between selected climatic variables and major wine-composition variables; Dataset S1 (Climate_Data): Climate indicators for the 16 winery–vintage observations, including annual, growing-season, and ripening-period temperature and precipitation variables and viticultural climate indices; Dataset S2 (Sensory_data): Sensory attribute intensity scores of Chardonnay wines from the Shandong Peninsula and the Chugoku region across the 2022 and 2023 vintages; Dataset S3 (OAV_data): Summed odor activity values (OAVs) for six aroma categories in Chardonnay wines across the two regions and vintages; Dataset S4 (Aroma_data): Volatile compound concentrations in Chardonnay wines across the two regions and vintages. Tables S1–S5 and Datasets S1–S4 are provided in a single Excel workbook.
